# Brazilian cohort study of risk factors associated with unsuccessful outcomes of drug resistant tuberculosis

**DOI:** 10.1186/s12879-021-06756-7

**Published:** 2021-10-09

**Authors:** Patricia Bartholomay, Rejane Sobrino Pinheiro, Fernanda Dockhorn, Daniele Maria Pelissari, Wildo Navegantes de Araújo

**Affiliations:** 1 Tropical Disease Post-Graduation Program, University of Brasilia, Brasília, DF Brazil; 2 Public Health Institute, Federal University of Rio de Janeiro, Rio de Janeiro, RJ Brazil; 3 National Tuberculosis Control Program, Health Surveillance Secretariat, Ministry of Health, Brasília, DF Brazil; 4 National Institute for Science and Technology for Health Technology Assessment, Porto Alegre, RS Brazil

**Keywords:** Tuberculosis, Drug resistance, Treatment, Outcomes

## Abstract

**Background:**

Treatment outcomes were evaluated of a cohort of new pulmonary tuberculosis (TB) cases that were rifampicin resistant, multidrug-resistant, or extensively resistant during 2013 and 2014 in Brazil. The objective of this study is to identify factors associated with unfavorable treatment outcomes for drug-resistant TB cases.

**Methods:**

The Brazilian Special Tuberculosis Treatment Information System (SITE-TB) was the main data source. The independent variables were classified into four blocks (block I: individual characteristics; block II: clinical characteristics and proposed treatment; block III: treatment follow-up characteristics; and block IV: TB history). The category of successful therapeutic outcome was compared with lost to follow-up, failure, and death. Considering the multiple outcomes as the dependent variable, the odds ratios (OR) and its respective 95% confidence interval (95% CI) were estimated by multinomial logistic regression.

**Results:**

After applying the exclusion criteria, 980 (98.8%) individuals were included in the study. Of these, 621 (63.4%) had successful treatment, 163 (16.6%) lost to follow-up, 76 (7.8%) failed, and 120 (12.2%) died. Important factors associated with lost to follow-up in the final model included use of illicit drugs (OR = 2.5 95% CI: 1.57–3.82). Outcome failure was associated with having disease in both lungs (OR = 2.0; 95% CI: 1.09–3.62) and using more than one or not using injectable medication (OR = 2.8; 95% CI: 1.05–7.69). Major factors for the death outcome were at least 60 years old (OR = 3.4; 95% CI: 1.90–6.03) and HIV positive (OR = 2.7; 95% CI: 1.45–4.83).

**Conclusions:**

The factors associated with unfavorable treatment outcomes were different. Some of these factors are specific to each outcome, which reflects the complexity of providing care to these individuals.

**Supplementary Information:**

The online version contains supplementary material available at 10.1186/s12879-021-06756-7.

## Introduction

Tuberculosis (TB) is one of the top 10 causes of death worldwide. In 2019, about 10 million people fell ill and 1.2 million died from TB [1]. According to Falzon et al. [2], multidrug-resistant (MDR) TB, which is defined as resistance to rifampicin (R) and isoniazid (H), plays a vital role in global TB control. In 2019 worldwide, 465 thousand people developed rifampicin resistant (RR) TB diagnosed by GeneXpert MTB/RIF® (RR) of which 78% had MDR-TB. Only 206 thousand (44%) were diagnosed and reported, and 177 thousand (38%) started treatment with second line drugs [1].

In Brazil, the strategies to contain the main causes associated with the development of drug-resistant TB (DR-TB) in the community include free treatment for all TB cases offered by the Unified Public Health System (SUS); standardization in a fixed-dose combination of treatments for sensitive TB and DR-TB; acquisition, supply, and distribution of centralized drugs, with quality control, guaranteed by the Brazilian Ministry of Health (MoH) and/or the World Health Organization (WHO). Added to this, the controlled availability of drugs to treat DR-TB cases requires validated of the regimen by medical specialists before their release [3]. While the worldwide estimate is 3.4% of new cases and 18% of retreatment cases have MDR/RR-TB, in Brazil the percentages are 1.5% and 8%, respectively [1]. The surveillance of MDR-TB started in the country in the 2000s, and in 2004, an online information system for notification of MDR-TB cases was created. In 2013, this system was implemented to report all known cases of DR-TB. These cases, when diagnosed in any health service, should be referred to treatment in a TB reference services. This care network also assists in the control of resistance to anti-TB drugs in Brazil.

Nevertheless, treatment outcomes and detection of MDR/RR-TB cases estimated by the WHO for the country are far from desirable. In Brazil, the percentage of detection did not exceed 63% in the last five years [1]. The management of DR-TB treatment is more complex than the sensitive regimen, requiring great effort from health professionals and patients to adhere to treatment.

The worse treatment outcome of DR-TB is associated with men, smoking, and HIV/AIDS [4]. The type of regime used [5] and adherence interventions, such as incentives, education, and digital technologies [6], have already been associated with treatment success. However, even with the increase in scientific articles on the topic, systematic reviews describe difficulties in data analysis, either due to incompleteness or methodological limitations [7–9].

As the Special Tuberculosis Treatment Information System (SITE-TB) was implemented only recently, studies using this system and evaluating treatment outcomes for DR-TB in Brazil remain scarce [5, 10]. Therefore, the objective of this study was to identify the factors associated with unfavorable treatment outcomes (lost to follow-up, failure, and death) for DR-TB cases.

## Methods

### Study design and population

This analytical study, of the historical cohort type, studied a population of new cases with pulmonary and mixed TB (pulmonary and extrapulmonary), starting treatment between 2013 and 2014 and with the following patterns of drug resistance at the beginning of treatment: RR-TB, MDR-TB, and TB with extensive resistance (XDR). All individuals included in the study had laboratory-confirmed tuberculosis. The diagnosis of resistance was made by sensitivity test or GeneXpert MTB/RIF®. Cases were excluded who were less than 15 years old, without definitive treatment reported, or were duplicated.

### Data source

The SITE-TB was the main source of data [11]. The history of TB treatment that occurred before the notification of SITE-TB was retrieved from the Information System on Notifiable Diseases (Sinan). The treatment outcomes were qualified with the death records in the Mortality Information System (SIM), through the probabilistic relationship of the database [12].

In Brazil, all TB cases must be reported in the Sinan. Cases diagnosed with DR-TB should be concluded in the Sinan and notified in the SITE-TB to monitor treatment [3]. Since the implementation of SITE-TB in 2013, all cases under treatment with special regimens, that is, different from the basic treatment regimen, must be recorded in this system. SITE-TB is an online information system. All information until the outcome is registered on the system, which makes it possible to follow patients even if they switch the health facility during the treatment [11].

### Study variables

Independent variables were classified into four blocks (block I: individual characteristics; block II: clinical characteristics and proposed treatment; block III: treatment follow-up characteristics; and block IV: TB history).

Individuals were divided into two age categories: 15 to 59 and over 59 years old. The ^“^no^”^ and ^“^do not know^”^ categories of the variables on diseases and associated conditions (alcoholism, diabetes, smoking, use of illicit drugs, and others) were grouped together forming the ^“^no/do not know^”^ category. Confirmation of alcoholism or smoking was defined by the physician. The use of illicit drugs was reported by the patient.

The HIV test variable was classified as yes, no, and missing (no test performed or result not recorded). Education was categorized into the following categories: up to seven years of education, eight or more years, and information not provided.

In block II, the initial scheme type was classified as standardized scheme (cases that started treatment using schemes define by the MoH recommendations [3]) and individualized scheme (for those cases that started treatment with any scheme different from the standardized schemes).

The fluoroquinolone treatment was defined as: used only levofloxacin or moxifloxacin, used only ofloxacin, and used more than one fluoroquinolone or did not use fluoroquinolone. The injectable drug treatment was categorized as: used only amikacin, used only streptomycin, used only capreomycin, and used more than one injectable or did not use injectable drugs. More than 80% of the cases identified with RR-TB also exhibited resistance to isoniazid, with the MDR resistance pattern confirmed after the sensitivity test [13]. Therefore, we classified the categories of the initial resistance pattern into MDR/RR and XDR.

In Block III, major adverse reactions were considered: auditory, mental, renal, or visual alterations; blood alterations; seizures; peripheral neuropathy; allergic reactions; vertigo; and nystagmus [14]. The minor adverse reactions considered included headache, skin hyperpigmentation, hyperuricemia, insomnia, gastrointestinal intolerance, nausea, and vomiting [14].

In Block IV, the previous tuberculosis events variable classified the number of previous tuberculosis events into two categories: up to three events and four or more events. The time between the first diagnosis of tuberculosis and the start of drug-resistant tuberculosis treatment was divided into three categories: up to one year, between one and three years, and three years or more.

The classification, categories, and description of each variable are provided in the Additional file 1: Table S1.

### Data analysis

To identify the factors associated with unfavorable outcomes in the treatment of DR-TB, records with unreported information were excluded when they represented less than 5% of all records. When the percentage was 5% or more, a category called ^“^unreported^”^ was created.

The outcome category ^“^success treatment^”^ was used as a reference for the variable response of treatment outcome and compared with the other categories (^“^lost to follow-up^”^, ^“^failure^”^, and ^“^death^”^). Cases with lost to follow-up are those who used the drugs for 30 days or more and stopped the treatment for 30 consecutive days or more. Failure was defined in the following situations: persistence of smear positive sputum at the end of treatment; cases who had strongly positive smear (+ + or +  + +) at the beginning of treatment and maintained this situation until the fourth month; initial positive smear followed by negative; and new positive results for two consecutive months, from the fourth month of treatment. Cases that died from tuberculosis or from other causes during the treatment were considered in the outcome death. For the multiple outcomes of the dependent variable, the odds ratios (OR) and their respective 95% confidence interval (95% CI) were estimated by multinomial logistic regression.

Initially, we included in the model all variables with a statistically significant association (α = 0.10) in the bivariate analysis of multinomial regression. Using the backward strategy, only the statistically significant variables were kept in the final model, considering the value of the significance level α = 0.05. The final model adequacy was evaluate with goodness-of-fit test by Hosmer–Lemeshow [15].

The final model was graphically represented by the OR logarithm and respective interval confidence of 95% (95%CI). The logarithm was chosen to represent the risk and protection factor on the same scale. A table with the OR, 95% CI and the p-value of the final model variables was also included. The project was approved by the Research Ethics Committee of the Faculdade de Medicina at the Universidade de Brasília (C.A.A.E 72432117.1.0000.5558 on 10/26/2017).

## Results

After excluding three cases under 15 years old, five duplicate records, and 17 records without the final information, 980 laboratory-confirmed cases of pulmonary RR/MDR/XDR-TB reported in the years 2013–2014 (98.8%) were included in the study (Additional file 1: Figure S2). Of these, 621 (63.4%) were successfully treated, 163 (16.6%) were lost to follow-up (LFU), 76 (7.8%) failed, and 120 (12.2%) died (Tables 1, 2, 3, 4).

**Table 1:** General distribution and outcome type of new cases of drug-resistant pulmonary tuberculosis, according to characteristics of block I, Brazil, 2013 and 2014

	Total (980 cases)		Success treatment		Lost to follow-up		Failure		Death		
	N	%	N	%	N	%	N	%		N	%
Block I											
*Sex*											
Male	652	66.5	412	63.2	120	18.4	46	7.1		74	11.3
Female	328	33.5	209	63.7	43	13.1	30	9.1		46	14.0
*Race/color*											
White	380	38.8	255	67.1	60	15.8	32	8.4		33	8.7
Brown/Black^a^	593	60.5	360	60.7	102	17.2	44	7.4		87	14.7
Asian/Indigenous	7	0.7	6	85.7	1	14.3	0	0.0		0	0.0
*Age group (years)*											
15–59	883	90.1	563	63.8	157	17.8	69	7.8		94	10.6
60 or over	97	9.9	58	59.8	6	6.2	7	7.2		26	26.8
*Education (years)*											
0 to 7	574	58.6	342	59.6	109	19.0	41	7.1		82	14.3
8 or more	318	32.4	229	72.0	36	11.3	31	9.7		22	6.9
Missing	88	9.0	50	56.8	18	20.5	4	4.5		16	18.2
*HIV*											
Positive	105	10.7	52	49.5	21	20.0	7	6.7		25	23.8
Negative	753	76.8	495	65.7	121	16.1	60	8.0		77	10.2
Missing	122	12.4	74	60.7	21	17.2	9	7.4		18	14.8
*Alcoholism*											
Yes	246	25.1	135	54.9	55	22.4	19	7.7		37	15.0
No/don't know	734	74.9	486	66.2	108	14.7	57	7.8		83	11.3
*Diabetes*											
Yes	144	14.7	98	68.1	14	9.7	10	6.9		22	15.3
No/don't know	836	85.3	523	62.6	149	17.8	66	7.9		98	11.7
*Smoking*											
Yes	241	24.6	139	57.7	47	19.5	18	7.5		37	15.4
No/don't know	739	75.4	482	65.2	116	15.7	58	7.8		83	11.2
*Use of illicit drugs*											
Yes	162	16.5	79	48.8	52	32.1	10	6.2		21	13.0
No/don't know	818	83.5	542	66.3	111	13.6	66	8.1		99	12.1
*Other associated diseases or conditions*^*b*^											
Yes	164	16.7	97	59.1	21	12.8	14	8.5		32	19.5
No/don't know	816	83.3	524	64.2	142	17.4	62	7.6		88	10.8
*Prison population*											
Yes	46	4.7	30	65.2	14	30.4	1	2.2		1	2.2
No	934	95.3	591	63.3	149	16.0	75	8.0		119	12.7
Total	980	100.0	621	63.4	163	16.6	76	7.8		120	12.2

^a^Brown/Black = combines black and brown

^b^Silicosis, neoplasms, transplant, user of TNF–alpha inhibitors and corticosteroids, seizure, viral hepatitis, renal failure/hemodialysis, and mental disorder

**Table 2:** General distribution and outcome type of new cases of drug-resistant tuberculosis, according to characteristics of block II, Brazil, 2013 and 2014

	Total (980 cases)		Success treatment		Lost to follow-up		Failure		Death	
	N	%	N	%	N	%	N	%	N	%
Block II										
*Cavitation*										
Yes	753	76.8	474	62.9	127	16.9	62	8.2	90	12.0
No	227	23.2	147	64.8	36	15.9	14	6.2	30	13.2
*Bilateral disease*										
Yes	614	62.7	369	60.1	101	16.4	57	9.3	87	14.2
No	366	37.3	252	68.9	62	16.9	19	5.2	33	9.0
*Resistance type*										
Primary	231	23.6	166	71.9	32	13.9	15	6.5	18	7.8
Acquired	749	76.4	455	60.7	131	17.5	61	8.1	102	13.6
*Initial resistance pattern*										
MDR/RR	965	98.5	616	63.8	162	16.8	74	7.7	113	11.7
XDR	15	1.5	5	33.3	1	6.7	2	13.3	7	46.7
*Initial scheme type*										
Individualized	873	89.1	558	63.9	142	16.3	66	7.6	107	12.3
Standardized	107	10.9	63	58.9	21	19.6	10	9.3	13	12.1
*Fluoroquinolone treatment*										
Only used Lfx or Mfx	935	95.4	594	63.5	158	16.9	70	7.5	113	12.1
Only used Ofx	12	1.2	6	50.0	3	25.0	2	16.7	1	8.3
Used more than one fluorquinolone or did not use	33	3.4	21	63.6	2	6.1	4	12.1	6	18.2
*Injectable drug treatment*										
Just used Am	242	24.7	151	62.4	41	16.9	17	7.0	33	13.6
Only used S	607	61.9	391	64.4	103	17.0	41	6.8	72	11.9
Only used Cm	49	5.0	30	61.2	10	20.4	5	10.2	4	8.2
Used more than one injectable or did not use	82	8.4	49	59.8	9	11.0	13	15.9	11	13.4
Total	980	100.0	621	63.4	163	16.6	76	7.8	120	12.2

*MDR* multidrug resistant, *RR* resistant to rifampicin diagnosed by GeneXpert MTB/RIF® (RR), *XDR* extensively resistant, *Lfx* levofloxacin, *Mfx* moxifloxacin, *Ofx* ofloxacino, *Am* amikacin, *S* streptomycin, *Cm* capreomycin

**Table 3:** General distribution and outcome type of new cases of drug-resistant pulmonary tuberculosis, according to characteristics of block III, Brazil, 2013 and 2014

	Total (980 cases)		Success treatment		Lost to follow-up		Failure		Death	
	N	%	N	%	N	%	N	%	N	%
Block III										
*Resides in different municipality than treatment*										
Yes	414	42.2	273	65.9	56	13.5	38	9.2	47	11.4
No	566	57.8	348	61.5	107	18.9	38	6.7	73	12.9
*Reported unfavorable clinical evaluation*										
Yes	215	21.9	75	34.9	49	22.8	52	24.2	39	18.1
No	765	78.1	546	71.4	114	14.9	24	3.1	81	10.6
*Changed regimen type*										
Yes	154	15.7	109	70.8	17	11.0	16	10.4	12	7.8
No	826	84.3	512	62.0	146	17.7	60	7.3	108	13.1
*Had adverse reaction*										
No adverse reaction records	757	77.2	459	60.6	142	18.8	57	7.5	99	13.1
Only minor adverse reactions	158	16.1	114	72.2	14	8.9	17	10.8	13	8.2
At least one major adverse reaction	65	6.6	48	73.8	7	10.8	2	3.1	8	12.3
Total	980	100.0	621	63.4	163	16.6	76	7.8	120	12.2

**Table 4:** General distribution and outcome type of new cases of drug-resistant pulmonary tuberculosis, according to characteristics of block IV, Brazil, 2013 and 2014

	Total (980 cases)		Success treatment		Lost to follow-up		Failure		Death	
	N	%	N	%	N	%	N	%	N	%
Block IV										
*Previous TB events*										
Up to 3 events	878	89.6	568	64.7	138	15.7	70	8.0	102	11.6
4 events or more	102	10.4	53	52.0	25	24.5	6	5.9	18	17.6
*Time between the first TB diagnosis and the onset of TB-DR treatment (years)*										
Up to 1	471	48.1	299	63.5	80	17.0	40	8.5	52	11.0
1 to 3	236	24.1	152	64.4	42	17.8	17	7.2	25	10.6
More than 3	273	27.9	170	62.3	41	15.0	19	7.0	43	15.8
Total	980	100.0	621	63.4	163	16.6	76	7.8	120	12.2

*TB* tuberculosis, *DR* drug resistant

The percentage of LFU was higher in men (18.4%) and the age group of 15–59 years (17.8%). For associated diseases, the percentage of LFU was higher in individuals with reported alcoholism (22.4%), no diabetes or missing information about diabetes (17.8%), smoking (19.5%), and use of illicit drugs (32.1%) (Table 1). For the variables in block II, the absence of cavitation (62.9%), bilateral disease (68.9%), primary resistance type (71.9%), and MDR/RR initial resistance pattern (63.8) presented the best treatment results (Table 2). In block III, the highest indicators for death were the same municipality of residence and treatment (12.9%), recorded unfavorable clinical evolution (18.1%), and no recorded regimen change (13.1%) (Table 3). Individuals who had up to three previous TB events registered on the Sinan exhibited 64.7% treatment success (Table 4). The nonadjusted model is in Additional file 1: Table S3.

**Fig. 1:**
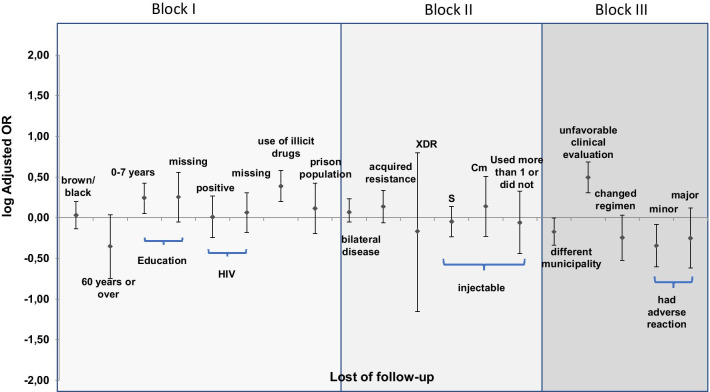
Graphical representation of the final model of multinomial logistic regression for factors associated with lost to follow-up in new cases of drug-resistant pulmonary tuberculosis. Brazil. 2013 and 2014. (980 cases). *XDR* extensively resistant, *S* streptomycin, *Cm* capreomycin

**Fig. 2:**
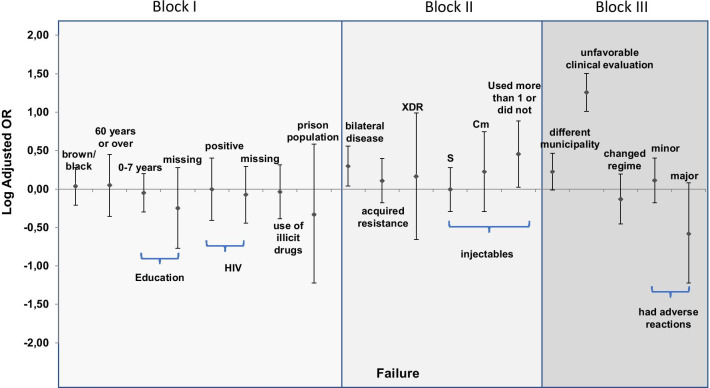
Graphical representation of the final model of multinomial logistic regression for factors associated with failure in new cases of drug-resistant pulmonary tuberculosis. Brazil. 2013 and 2014. (980 cases). *XDR* extensively resistant, *S* streptomycin, *Cm* capreomycin

**Fig. 3:**
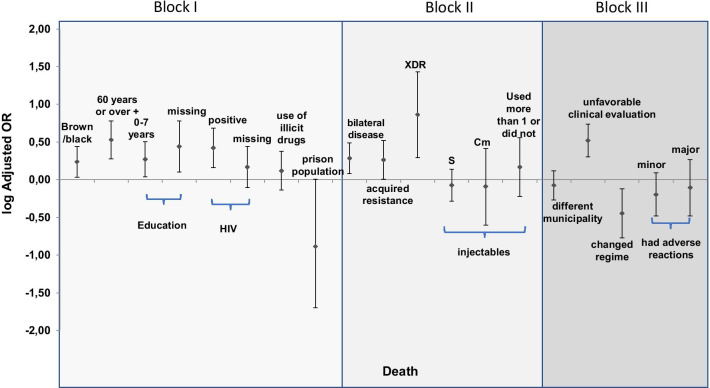
Graphical representation of the final model of multinomial logistic regression for factors associated with death in new cases of drug-resistant pulmonary tuberculosis. Brazil. 2013 and 2014. (980 cases). *XDR* extensively resistant, *S* streptomycin, *Cm* capreomycin

The final model is presented in Figs. 1, 2, and 3 with OR logarithm and respective CI for each outcome. Variables whose confidence intervals do not cross the null value are statistically significant. The results of the final model (OR and IC) are also provided in Table 5.

**Table 5:** Final model resulting from multinomial logistic regression for factors associated with unfavorable outcomes in new cases of drug-resistant pulmonary tuberculosis, Brazil, 2013 and 2014. (980 cases)

	Lost to follow-up			Failure			Death		
	Adjusted OR	(CI95%)	p value*	Adjusted OR	(CI95%)	p value*	Adjusted OR	(CI95%)	p value*
Block I									
*Race/color*									
White	1.0			1.0			1.0		
Brown/Black1	1.1	(0.73–1.58)	0.72	1.1	(0.62–1.90)	0.76	1.7	(1.08–2.77)	0.02
Asian/Indigenous	0.8	(0.08–6.77)	0.80			0.99			0.99
*Age group (years)*									
15–59	1.0			1.0			1.0		
60 or more	0.4	(0.18–1.09)	0.08	1.1	(0.44–2.82)	0.81	3.4	(1.90–6.03)	< 0.01
*Education (years)*									
0 to 7	1.8	(1.13–2.66)	0.01	0.9	(0.50–1.59)	0.69	1.9	(1.09–3.19)	0.02
8 or more	1.0			1.0			1.0		
Missing	1.8	(0.89–3.62)	0.10	0.6	(0.17–1.90)	0.36	2.8	(1.27–6.02)	0.01
*HIV*									
Positive	1.0	(0.57–1.85)	0.94	1.0	(0.39–2.52)	0.99	2.7	(1.45–4.83)	< 0.01
Negative	1.0			1.0			1.0		
Missing	1.2	(0.66–2.02)	0.61	0.8	(0.36–1.97)	0.70	1.5	(0.79–2.76)	0.22
*Use of illicit drugs*									
Yes	2.5	(1.57–3.82)	< 0.01	0.9	(0.41–2.06)	0.84	1.3	(0.73–2.38)	0.37
No/don't know	1.0			1.0			1.0		
*Prison population*									
Yes	1.3	(0.64–2.65)	0.48	0.5	(0.06–3.85)	0.48	0.1	(0.02–1.01)	0.05
No/don't know	1.0			1.0			1.0		
Block II									
*Bilateral disease*									
Yes	1.2	(0.89–1.71)	0.43	2.0	(1.09–3.62)	0.03	1.9	(1.20–3.08)	0.01
No	1.0			1.0			1.0		
*Resistance type*									
Primary	1.0			1.0			1.0		
Acquired	1.4	(0.87–2.17)	0.17	1.3	(0.66–2.50)	0.47	1.8	(1.02–3.31)	0.04
*Initial resistance pattern*									
MDR/RR	1.0			1.0			1.0		
XDR	0.7	(0.07–6.25)	0.73	1.5	(0.22–9.69)	0.70	7.3	(1.97–27.09)	< 0.01
*Injectable drug treatment*									
Only used Am	1.0			1.0			1.0		
Only used S	0.9	(0.58–1.38)	0.61	1.0	(0.51–1.90)	0.97	0.8	(0.52–1.38)	0.50
Only use Cm	1.4	(0.59–3.24)	0.46	1.7	(0.51–5.55)	0.40	0.8	(0.25–2.61)	0.73
Used more than one injectable or did not use	0.9	(0.36–2.13)	0.76	2.8	(1.05–7.69)	0.04	1.5	(0.60–3.61)	0.40
Block III									
*Resides in different municipality than treatment*									
Yes	0.7	(0.46–0.99)	0.04	1.7	(0.97–2.92)	0.07	0.8	(0.54–1.31)	0.45
No	1.0			1.0			1.0		
*Reported unfavorable clinical evaluation*									
Yes	3.1	(2.02–4.86)	< 0.01	18.0	(10.18–31.90)	< 0.01	3.3	(2.02–5.44)	< 0.01
No	1.0			1.0			1.0		
*Changed regimen type*									
Yes	0.6	(0.30–1.07)	0.08	0.7	(0.35–1.57)	0.43	0.4	(0.17–0.76)	0.01
No	1.0			1.0			1.0		
*Had adverse reaction*									
No adverse reaction records	1.0			1.0			1.0		
Only minor adverse reactions	0.5	(0.25–0.83)	0.01	1.3	(0.66–2.52)	0.45	0.6	(0.33–1.23)	0.18
At least one major adverse reaction	0.6	(0.24–1.32)	0.18	0.3	(0.06–1.21)	0.09	0.8	(0.33–1.85)	0.58

IBlack = combines black and brown

*OR* odds ratio, *CI* confidence interval, *MDR* multidrug resistant, *RR* resistant to rifampicin diagnosed by GeneXpert MTB/RIF® (RR), *XDR* extensively resistant, *Am* amikacin, *S* streptomycin, *Cm* capreomycin

*Significance level = 0.05

In the final model, a greater chance of LFU was associated with less than eight years of education (OR = 1.8; 95% CI: 1.13–2.66), use of illicit drugs (OR = 2.5 95% CI: 1.57–3.82), and report unfavorable clinical evolution (OR = 3.1; 95% CI: 2.02–4.86). Living in a different municipality from the treatment site (0.7; 95% CI: 0.46–0.99) and only minor adverse reactions recorded compared to no adverse reactions recorded (OR = 0.5; 95% CI: 0.25–0.83) were protective factors against LFU in the bivariate analysis (Fig. 1 and Table 5).

Exhibiting disease in both lungs (OR = 2.0; 95% CI: 1.09–3.62), using more than one or not using an injectable drug compared to the use of amikacin (OR = 2.8; 95% CI: 1.05–7.69), and reporting unfavorable clinical evolution (OR = 18.0; 95% CI: 10.18–31.90) were associated with failure (Fig. 2 and Table 5).

The case had a greater chance of ending in death when the individual was brown/black (OR = 1.7; 95% CI: 1.08–2.77), 60 years or older (OR = 3.4; 95% CI: 1.90–6.03), zero to seven years of education (OR = 1.9; 95% CI 1.09–3.19), or missing education information (OR = 2.8; 95% CI: 1.27–6.02), HIV positive (OR = 2.7; 95% CI: 1.45–4.83), disease affecting both lungs (OR = 1.9; 95% CI: 1.20–3.08), acquired resistance type (OR = 1.8; 95% CI: 1.02–3.31), XDR resistance (OR = 7.3; 95% CI: 1.97–27.09), and notified unfavorable clinical evolution (OR = 3.3; 95% CI: 2.02–5.44). On the other hand, change in treatment regimen due to a major adverse reaction or increased the resistance pattern was a protective factor (OR = 0.4; 95% CI 0.17–0.76) (Fig. 3 and Table 5). The table with the final adjusted model is in Additional file 1: Table S4.

## Discussion

The treatment success for individuals in the study cohort (63.4%) was higher than that achieved worldwide (55%) in 2016 for cases of MDR/RR-TB; however, it was still far short of the values considered acceptable by the WHO [1]. Our analysis evidenced that the factors related to the unfavorable outcomes studied are distinct and multifactorial. Individual and follow-up treatment factors were related to LFU. For failure, the clinical characteristics, proposed treatment, and follow-up stand out. For death, issues related to individual, clinical, treatment characteristics, and follow-up treatment are related to this outcome. The protective effect against LFU of recorded minor adverse reactions compared to no recorded adverse reactions suggests that factors related to the quality of health services, which were not included in the study, may also influence unfavorable outcomes of DR-TB. The hypothesis is that health units with professionals that were more sensitive to user complaints, where the presence of minor adverse reactions is valued, obtained better treatment outcomes.

Low education was associated with LFU and death. Several studies have already associated education with adherence to sensitive and MDR-TB treatment [10, 16–18]. The significance of this finding can be extrapolated to their conditions, as education is recognized as a proxy of socioeconomic status [19]. The same can be said for brown/black race/color [16,20], which was also associated with death. Research has already proven that the mitigation of social determinants is essential to control TB [21]. However, for DR-TB, this is not enough for therapeutic success. A study that assessed the impact on the outcome of DR-TB of social and economic intervention demonstrated satisfactory results for adherence but no impact on treatment failure [22].

Having lesions detected in both lungs suggests a late diagnosis and this factor was related to failure and death, as was also found in other studies [18, 23, 24]. In the study period, the GeneXpert MTB/RIF® was just being implemented in Brazil and culture was taken for 26% and 42% for new and retreatment cases, respectively [25]. These results confirm the difficulty in making the diagnosis of MDR/RR-TB and corroborates the hypothesis that individuals arrived too late for treatment, influencing therapeutic success. The GeneXpert MTB/RIF® established in Brazil can diagnose 57% of new TB cases; however, only 33% of new cases in the country performed GeneXpert MTB/RIF® [25]. These results suggest that the available laboratory network is still not suitable for sending samples to laboratories with equipment or that the TB diagnostic algorithm is not being complied with according to national recommendations, making access to the exam more difficult [3]. The protective association against LFU of living in a municipality other than the treatment location also may indicate that access to DR-TB diagnosis and treatment may have influenced the outcome. The individuals who obtain DR-TB diagnosis in smaller municipalities, without TB reference services are probably different from those who live in large cities, where access to diagnosis DR-TB is broader.

Alcoholism has already been associated with the worst outcomes of sensitive and MDR-TB [9, 16, 26, 27]. This same association occurred in the bivariate analysis of this study for LFU and death, but it was not maintained in the final model. The inclusion in the final model of variables related to alcoholism, including illicit drug use and education, may explain why alcoholism did not remain associated. A study that evaluated the relationship between alcohol consumption and the outcome of MDR-TB treatment concluded that this consumption may be an indicator for other behavioral disorders [27], such as illicit drug use, which maintained the association with LFU in the final model. Another factor that may have influenced this result is the lack of a standardized definition of alcoholism in the TB notification forms.

The gender difference was not found to influence the DR-TB treatment outcomes assessed in this study, which corroborates other studies carried out in Brazil and in other BRICS countries [10, 27, 28]. However, studies about sensitive tuberculosis have already associated males with less chances for cure, which could be because men seek health services less often and how the health services organize their programmatic activities, prioritizing the maternal and child population [29, 30].

The association between the XDR resistance pattern and death was expected [3]. With the launch in 2019 of the TB Guidelines in Brazil, the MoH reinforced the need for early diagnosis of these cases, recommending considering failure of the first treatment of tuberculosis to be MDR-TB when there is no bacteriological conversion and no clinical improvement after eight months of treatment. In addition, these Guidelines also standardize the therapeutic regimen for XDR-TB, which before publication was only performed individually [3].

The final multivariate model associated HIV positivity with death. Studies have already found this result [9, 31, 32], as well as the greater occurrence of adverse effects and drug interactions in these cases [33]. The early diagnosis of drug resistance, the appropriate definition of the treatment, and the rapid initiation of antiretroviral therapy (ART) two weeks after starting TB treatment, if the patient is not already on ART, are recommendations for the treatment of co-infected patients [33, 34].

Having reported unfavorable clinical evolution at any time during treatment was strongly associated with all the unfavorable outcomes studied. This information is included in SITE-TB when the physician responsible for the follow-up appointments observe a worsening of clinical symptoms, imaging, or laboratory tests. Thus, this information can be used by professionals who monitor patients with DR-TB as a sign of the clinical evolution of a potential unfavorable outcome during treatment.

This study has some limitations in addition to those inherent to retrospective observational studies based on secondary databases. Few patients had undergone a sensitivity test for second-line drugs, which may have caused classification bias of the initial resistance pattern and the change in the resistance pattern of the cases during the treatment. Another limitation was the way in which SITE-TB registers associated diseases and conditions as well as adverse reactions, not differentiating missing data from the absence of the disease/condition/adverse reaction, generating a possible classification bias and underestimating the occurrence of these factors. The variable HIV result does not have this limitation; however, for the HIV and the education variables, the option ^“^missing^”^ among the analysis categories was necessary. In addition, there is no standardized definition of smoking and alcoholism for filling out the notification forms. The SITE-TB does not allow exporting the treatment scheme used in the case of individualized schemes. Therefore, it was only possible to know the drugs used in each case, which was included in the analyzes.

The exclusion of 17 records (1.7%), given the type of conclusion completed at the time of exporting the database, may have influenced the results. Likewise, 774 (88.1%) MDR-TB cases and 104 (11.9%) RR-TB cases were included as a single category of initial resistance pattern and this categorization may also have influenced our findings. Finally, the unavailability of the body mass index, also predictors of unfavorable outcomes already studied [35], could be a limitation.

## Conclusions

In the historical cohort of DR-TB case analyses, the factors associated with unfavorable treatment outcomes were different. Some factors were specific to each outcome, which reflects the complexity of the care provided to these individuals.

These results may be used in the development of an index to identify the risk of unsuccessful outcomes for DR-TB cases, helping services to identify cases with a greater chance of unfavorable treatment outcomes. Although the WHO recommends short or long-term regimens, including bedaquiline and pretomanid, to facilitate adherence to MDR-TB treatment, these are not yet available in Brazil [3, 36]. The implementation of these new technologies may provide benefits to the therapeutic success of DR-TB and modify the results found. Implementation of DR-TB diagnosis, with wide access to molecular tests to detect antimicrobial resistance markers and to first- and second-line sensitivity tests, is another factor that could assist in the outcome of cases by permitting early diagnosis of anti-TB drug resistance. Finally, studies should be conducted that include in the analyzes the association between the quality of TB reference services, the phenotypic diversity of strains in the cases, and the outcomes of DR-TB.

## Supplementary Information

**Additional file 1: Table S1.** Table with description of the independent variables used in the study. **Figure S2.** Flowchart for selection of the studied population. **Table S3.** Not adjusted odds ratio of each unfavorable outcome for new cases of drug-resistant pulmonary tuberculosis, according to studied variables, Brazil, 2013 and 2014 (980 cases). **Table S4.** Table with goodness-of-fit test for a final multinomial logistic regression model result.

## Data Availability

Restrictions apply to the availability of these data, which were used under license for the current study, and so are not publicly available. However, the data are available from the authors upon reasonable request.

## Abbreviations

TB: Tuberculosis
MDR: Multidrug-resistant
RR: Rifampicin resistant
DR: Drug-resistant
SUS: Unified Public Health System
MoH: Ministry of Health
WHO: World Health Organization
SITE-TB: Special Tuberculosis Treatment Information System
XDR: Extensive resistance
Sinan: Information system on notifiable diseases
SIM: Mortality Information System
OR: Odds ratios
CI: Confidence interval
LFU: Lost to follow-up
Lfx: Levofloxacin
Mfx: Moxifloxacin
Ofx: Ofloxacino
Am: Amikacin
S: Streptomycin
Cm: Capreomycin
ART: Antiretroviral therapy

## References

[1] WHO. Global tuberculosis report 2020. Geneva: World Health Organization; 2020.

[2] Falzon D, Mirzayev F, Wares F, Baena IG, Zignol M, Linh N (2015). Multidrug-resistant tuberculosis around the world: what progress has been made?. Eur Respir J.

[3] Brasil. Manual de recomendações para o controle da tuberculose no Brasil. 2a edição. Brasília: Ministério da Saúde. Secretaria de Vigilância em Saúde. Departamento de Vigilância Epidemiológica; 2019.

[4] Limenih YA, Workie DL (2019). Survival analysis of time to cure on multi-drug resistance tuberculosis patients in Amhara region, Ethiopia. BMC Public Health.

[5] Bastos ML, Cosme LB, Fregona G, do Prado TN, Bertolde AI, Zandonade E (2017). Treatment outcomes of MDR-tuberculosis patients in Brazil: a retrospective cohort analysis. BMC Infect Dis.

[6] Alipanah N, Jarlsberg L, Miller C, Linh NN, Falzon D, Jaramillo E (2018). Adherence interventions and outcomes of tuberculosis treatment: a systematic review and meta-analysis of trials and observational studies. PLoS Med.

[7] Bastos ML, Lan Z, Menzies D (2017). An updated systematic review and meta-analysis for treatment of multidrug-resistant tuberculosis. Eur Respir J.

[8] Ho J, Byrne AL, Linh NN, Jaramillo E, Fox GJ (2017). Decentralized care for multidrug-resistant tuberculosis: a systematic review and meta-analysis. Bull World Health Organ.

[9] Samuels JP, Sood A, Campbell JR, Ahmad Khan F, Johnston JC (2018). Comorbidities and treatment outcomes in multidrug resistant tuberculosis: a systematic review and meta-analysis. Sci Rep.

[10] Viana PV de S, Redner P, Ramos JP. Fatores associados ao abandono e ao óbito de casos de tuberculose drogarresistente (TBDR) atendidos em um centro de referência no Rio de Janeiro, Brasil. Cad Saúde Pública. 2018. 34(5). Disponível em: http://www.scielo.br/scielo.php?script=sci_arttext&pid=S0102-311X2018000505005&lng=pt&tlng=pt. Accessed 14 May 2019. 10.1590/0102-311X00048217 29768580

[11] Bartholomay P, Pinheiro RS, Pelissari DM, Arakaki-Sanchez D, Dockhorn F, Rocha JL (2019). Sistema de Informação de Tratamentos Especiais de Tuberculose (SITE-TB): histórico, descrição e perspectivas. Epidemiol E Serviços Saúde..

[12] Rocha MS, Oliveira GP de, Guillen LCT, Coeli CM, Saraceni V, Pinheiro RS, et al. Uso de linkage entre diferentes bases de dados para qualificação de variáveis do Sinan-TB e a partir de regras de scripting. Cad Saúde Pública. 2019;35(12). Disponível em: http://www.scielo.br/scielo.php?script=sci_abstract&pid=S0102-311X2019001404001&lng=en&nrm=iso&tlng=pt. Accessed 8 Aug 2020. 10.1590/0102-311X00074318 31800783

[13] Durovni B, Saraceni V, van den Hof S, Trajman A, Cordeiro-Santos M, Cavalcante S (2014). Impact of replacing smear microscopy with Xpert MTB/RIF for diagnosing tuberculosis in Brazil: a stepped-wedge cluster-randomized trial. PLOS Med.

[14] Arbex MA, Varella M de CL, Siqueira HR de, Mello FAF de. Antituberculosis drugs: drug interactions, adverse effects, and use in special situations. Part 2: second line drugs. J Bras Pneumol Publicaça̋o. 2010;36(5):641–56. 10.1590/s1806-37132010000500017 21085831

[15] Hosmer DW, Lemeshow S (1980). Goodness-of-fit tests for the multiple logistic regression model. Commun Stat Methods.

[16] do Prado TN, Rajan JV, Miranda AE, Dias EdS, Cosme LB, Possuelo LG (2017). Clinical and epidemiological characteristics associated with unfavorable tuberculosis treatment outcomes in TB-HIV co-infected patients in Brazil: a hierarchical polytomous analysis. Braz J Infect Dis.

[17] Tupasi TE, Garfin AMCG, Kurbatova EV, Mangan JM, Orillaza-Chi R, Naval LC (2016). Factors associated with loss to follow-up during treatment for multidrug-resistant tuberculosis, the Philippines, 2012–2014. Emerg Infect Dis.

[18] Bhering M, Kritski A (2020). Primary and acquired multidrug-resistant tuberculosis: Predictive factors for unfavorable treatment outcomes in Rio de Janeiro, 2000–2016. Rev Panam Salud Pública..

[19] Mackenbach JP, Kunst AE (1997). Measuring the magnitude of socio-economic inequalities in health: an overview of available measures illustrated with two examples from Europe. Soc Sci Med 1982..

[20] Oliveira BLCA de, Luiz RR, Oliveira BLCA de, Luiz RR. Densidade racial e a situação socioeconômica, demográfica e de saúde nas cidades brasileiras em 2000 e 2010. Rev Bras Epidemiol. 2019. 22 Disponível em: http://www.scielo.br/scielo.php?script=sci_abstract&pid=S1415-790X2019000100432&lng=en&nrm=iso&tlng=pt. Accessed 24 Jun 2019. 10.1590/1980-549720190036 31038617

[21] Lönnroth K, Castro KG, Chakaya JM, Chauhan LS, Floyd K, Glaziou P (2010). Tuberculosis control and elimination 2010–50: cure, care, and social development. Lancet.

[22] Bhatt R, Chopra K, Vashisht R (2019). Impact of integrated psycho-socio-economic support on treatment outcome in drug resistant tuberculosis—a retrospective cohort study. Indian J Tuberc.

[23] Gegia M, Kalandadze I, Madzgharashvili M, Furin J (2011). Developing a human rights-based program for tuberculosis control in Georgian prisons. Health Hum Rights.

[24] Leimane V, Dravniece G, Riekstina V, Sture I, Kammerer S, Chen MP (2010). Treatment outcome of multidrug/extensively drug-resistant tuberculosis in Latvia, 2000–2004. Eur Respir J.

[25] Brasil. Tabnet. Informações de saúde. 2019. Disponível em: http://tabnet.datasus.gov.br/cgi/tabcgi.exe?sinannet/cnv/tubercbr.def. Accessed 6 Mar 2019.

[26] Garrido MdS, Penna ML, Perez-Porcuna TM, de Souza AB, Marreiro LdS, Albuquerque BC (2012). Factors associated with tuberculosis treatment default in an endemic area of the Brazilian Amazon: a case control-study. PLoS ONE.

[27] Duraisamy K, Mrithyunjayan S, Ghosh S, Nair SA, Balakrishnan S, Subramoniapillai J (2014). Does alcohol consumption during multidrug-resistant tuberculosis treatment affect outcome? A population-based study in Kerala, India. Ann Am Thorac Soc.

[28] Wang J, Pang Y, Jing W, Chen W, Guo R, Han X (2019). Efficacy and safety of cycloserine-containing regimens in the treatment of multidrug-resistant tuberculosis: a nationwide retrospective cohort study in China. Infect Drug Resist.

[29] Braga JU, Pinheiro J dos S, Matsuda J da S, Barreto JAP, Feijão AMM. Fatores associados ao abandono do tratamento da tuberculose nos serviços de atenção básica em dois municípios brasileiros, Manaus e Fortaleza, 2006 a 2008. Cad Saúde Colet Rio J. 2012. 20(2) Disponível em: http://bases.bireme.br/cgi-bin/wxislind.exe/iah/online/?IsisScript=iah/iah.xis&src=google&base=LILACS&lang=p&nextAction=lnk&exprSearch=644855&indexSearch=ID. Accessed 30 Jul 2019.

[30] Pinheiro RS, Viacava F, Travassos C, Brito AdS (2002). Gênero, morbidade, acesso e utilização de serviços de saúde no Brasil. Ciênc Amp Saúde Coletiva..

[31] Farley JE, Ram M, Pan W, Waldman S, Cassell GH, Chaisson RE (2011). Outcomes of multi-drug resistant tuberculosis (MDR-TB) among a cohort of South African patients with high HIV prevalence. PLoS ONE.

[32] Girum T, Muktar E, Lentiro K, Wondiye H, Shewangizaw M (2018). Epidemiology of multidrug-resistant tuberculosis (MDR-TB) in Ethiopia: a systematic review and meta-analysis of the prevalence, determinants and treatment outcome. Trop Dis Travel Med Vaccines.

[33] Seung KJ, Keshavjee S, Rich ML (2015). Multidrug-resistant tuberculosis and extensively drug-resistant tuberculosis. Cold Spring Harb Perspect Med.

[34] Bhering M, Duarte R, Kritski A (2021). Treatment outcomes and predictive factors for multidrug-resistant TB and HIV coinfection in Rio de Janeiro State, Brazil. Int J Tuberc Lung Dis.

[35] Gler MT, Guilatco R, Caoili JC, Ershova J, Cegielski P, Johnson JL (2013). Weight gain and response to treatment for multidrug-resistant tuberculosis. Am J Trop Med Hyg.

[36] WHO. WHO consolidated guidelines on drug-resistant tuberculosis treatment. Geneva: World Health Organization; 2019. (End TB Strategic). 30946559

