# Supplementary material for: Brazilian cohort study of risk factors associated with unsuccessful outcomes of drug resistant tuberculosis
Source: BMC Infect Dis. 2021 Oct 9;21:1049. doi: 10.1186/s12879-021-06756-7 (PMC8502313; doi:10.1186/s12879-021-06756-7)
Supplement: Supplementary file 1 — Additional file 1: Table S1. Table with description of the independent variables used in the study. Figure S2. Flowchart for selection of the studied population. Table S3. Not adjusted odds ratio of each unfavorable outcome for new cases of drug-resistant pulmonary tuberculosis, according to studied variables, Brazil, 2013 and 2014 (980 cases). Table S4. Table with goodness-of-fit test for a final multinomial logistic regression model result. [file 12879_2021_6756_MOESM1_ESM.docx]

##### Additional material

Table S1: Table with description of the independent variables used in the study

| Block and Variable |  | Categories | Observations |
| --- | --- | --- | --- |
| Block I – individual characteristics |  |  |  |
| Sex |  | male and female |  |
| Race/skin color |  | white; brown/black and Asian/indigenous |  |
| Age range |  | 15–59 years and 60 or more years |  |
| Education |  | from 0 to 7 years of study; 8 or more years of study and missing |  |
| HIV |  | positive; negative; and missing |  |
| Alcoholism |  | yes and no/don't know |  |
| Diabetes |  | yes and no/don't know |  |
| Smoking |  | yes and no/don't know |  |
| Use of illicit drugs |  | yes and no/don't know |  |
| Other associated diseases and conditions |  | yes and no/don't know | This variable includes silicosis, Neoplasms, transplant, user of TNF alpha inhibitors and corticosteroids, seizure, viral hepatitis, renal failure/hemodialysis, and mental disorder |
| Prison population |  | Yes and no | This data was extracted from the local variable of probable contagion |
| Block II – clinical characteristics and proposed treatment |  |  |  |
| Cavitation |  | Yes and no |  |
| Bilateral disease |  | Yes and no |  |
| Resistance type |  | primary and acquired | Individuals with a record of the type of primary resistance at SITE–TB but with more than one previous notification of tuberculosis in Sinan, had the type of resistance changed to acquired. |
| Initial resistance pattern |  | Resistance to rifampicin by GeneXpert MTB/RIF®(RR)/resistance to rifampicin and isoniazid (MDR) and resistance to rifampicin and isoniazid plus resistance to fluoroquinolone and second line injectables (XDR) |  |
| Initial scheme type |  | standardized and individualized |  |
| Fluoroquinolone treatment |  | Used only levofloxacin or moxifloxacin; used only ofloxacin; and used more than one fluoroquinolone or did not use fluoroquinolone |  |
| Injectable drug treatment |  | Used only amikacin; used only streptomycin; used only capreomycin; and used more than one injectable or did not use injectable |  |
| Block III – Treatment follow–up characteristics |  |  |  |
| Resides in different municipality than treatment |  | Yes and no |  |
| Reported unfavorable clinical course |  | Yes and no | May have been informed at any time during the treatment |
| Changed regimen type |  | Yes and no | Marked as yes when the current treatment is different from the initial one. It was not possible to identify changes between individualized regimens in both moments |
| Had adverse reaction |  | There was no adverse reaction; only minor adverse reactions recorded; record of at least one major adverse reaction |  |
| Block IV – history of tuberculosis |  |  | The definition of the categories for the two variables in block IV accounted for the distribution of the records and the arbitrariness of the authors |
| Previous TB events |  | Up to 3 records and 4 or more records | All records found in Sinan prior to the start date of treatment at SITE–TB were added. |
| Time between the first diagnosis of tuberculosis and the start of drug-resistant tuberculosis treatment |  | Up to 1 year; between 1 and 3 years; and 3 years or more |  |

Figure S2: Flowchart for selection of the studied population

TB: tuberculosis

RR: resistant to rifampicin diagnosed by GeneXpert MTB/RIF®

MDR: multidrug resistant

XDR: extensively resistant

SITE-TB: Sistema de Informação de Tratamentos Especiais de Tuberculose

DR: drug resistant

Sinan: Sistema de Informação de Agravos de Notificação

SIM: Sistema de Informações sobre Mortalidade

^1^cure e complete treatment

Table S3: Not adjusted odds ratio of each unfavorable outcome for new cases of drug-resistant pulmonary tuberculosis, according to studied variables, Brazil, 2013 and 2014, (980 cases)

|  | Lost to follow-up | | | | Failure | | | | Death | | | | |  |
| --- | --- | --- | --- | --- | --- | --- | --- | --- | --- | --- | --- | --- | --- | --- |
|  | Not adjusted OR | | (CI95%) | p value* | Not adjusted OR | | (CI95%) | p value* | | Not adjusted OR | | (CI95%) | p value* | |
| Block I |  |  | |  |  |  | |  | |  |  | |  | |
| Sex |  |  | |  |  |  | |  | |  |  | |  | |
| Male | 1.4 | (0.96–2.08) | | **0.08** | 0.8 | (0.48–1.27) | | 0.31 | | 0.8 | (0.54–1.22) | | 0.32 | |
| Female | 1.0 | . | | . | 1.0 | . | | . | | 1.0 | . | | . | |
| Race/color |  |  | |  |  |  | |  | |  |  | |  | |
| White | 1.0 | . | | . | 1.0 | . | | . | | 1.0 | . | | . | |
| Brown/Black^1^ | 1.2 | (0.84–1.72) | | 0.31 | 1.0 | (0.60–1.58) | | 0.92 | | 1.9 | (1.21–2.88) | | **0.01** | |
| Asian/Indigenous | 0.7 | (0.08–5.99) | | 0.75 | . | . | | 0.99 | | . | . | | 0.99 | |
| Age group (years) |  |  | |  |  |  | |  | |  |  | |  | |
| 15–59 | 1.0 | . | | . | 1.0 | . | | . | | 1.0 | . | | . | |
| 60 or over | 0.4 | (0.16–0.88) | | **0.02** | 1.0 | (0.43–2.24) | | 0.97 | | 2.7 | (1.61–4.48) | | **<0.01** | |
| **Education (years)** |  |  | |  |  |  | |  | |  |  | |  | |
| 0 to 7 | 2.0 | (1.34–3.06) | | **<0.01** | 0.9 | (0.54–1.45) | | 0.63 | | 2.5 | (1.51–4.11) | | **<0.01** | |
| 8 or more | 1.0 | . | | . | 1.0 | . | | . | | 1.0 | . | | . | |
| Missing | 2.3 | (1.20–4.36) | | **0.01** | 0.6 | (0.20–1.75) | | 0.34 | | 3.3 | (1.63–6.80) | | **<0.01** | |
| **HIV** |  |  | |  |  |  | |  | |  |  | |  | |
| Positive | 1.7 | (0.96–2.85) | | **0.07** | 1.1 | (0.48–2.56) | | 0.81 | | 3.1 | (1.81–5.27) | | **<0.01** | |
| Negative | 1.0 | . | | . | 1.0 | . | | . | | 1.0 | . | | . | |
| Missing | 1.2 | (0.69–1.96) | | 0.58 | 1.0 | (0.48–2.11) | | 0.99 | | 1.6 | (0.89–2.76) | | 0.12 | |
| Alcoholism |  |  | |  |  |  | |  | |  |  | |  | |
| Yes | 1.8 | (1.26–2.67) | | **<0.01** | 1.2 | (0.69–2.09) | | 0.52 | | 1.6 | (1.04–2.47) | | **0.03** | |
| No/don't know | 1.0 | . | | . | 1.0 | . | | . | | 1.0 | . | | . | |
| **Diabetes** |  |  | |  |  |  | |  | |  |  | |  | |
| Yes | 0.5 | (0.28–0.90) | | **0.02** | 0.8 | (0.40–1.63) | | 0.55 | | 1.2 | (0.72–2.00) | | 0.49 | |
| No/don't know | 1.0 | . | | . | 1.0 | . | | . | | 1.0 | . | | . | |
| **Smokes** |  |  | |  |  |  | |  | |  |  | |  | |
| Yes | 1.4 | (0.95–2.07) | | **0.09** | 1.1 | (0.61–1.89) | | 0.80 | | 1.5 | (1.00–2.38) | | **0.05** | |
| No/don't know | 1.0 | . | | . | 1.0 | . | | . | | 1.0 | . | | . | |
| **Use of illicit drugs** |  |  | |  |  |  | |  | |  |  | |  | |
| Yes | 3.2 | (2.14–4.82) | | **<0.01** | 1.0 | (0.51–2.11) | | 0.91 | | 1.5 | (0.86–2.46) | | 0.16 | |
| No/don't know | 1.0 | . | | . | 1.0 | . | | . | | 1.0 | . | | . | |
| **Other associated diseases or conditions^2^** |  |  | |  |  |  | |  | |  |  | |  | |
| Yes | 0.8 | (0.48–1.33) | | 0.39 | 1.2 | (0.66–2.27) | | 0.53 | | 2.0 | (1.24–3.11) | | **<0.01** | |
| No/don't know | 1.0 | . | | . | 1.0 | . | | . | | 1.0 | . | | . | |
| **Prison Population** |  |  | |  |  |  | |  | |  |  | |  | |
| Yes | 1.9 | (0.96–3.58) | | **0.07** | 0.3 | (0.04–1.95) | | 0.19 | | 0.2 | (0.02–1.23) | | **0.08** | |
| No | 1.0 | . | | . | 1.0 | . | | . | | 1.0 | . | | . | |
| Block II |  |  | |  |  |  | |  | |  |  | |  | |
| Cavitation |  |  | |  |  |  | |  | |  |  | |  | |
| Yes | 1.1 | (0.72–1.65) | | 0.67 | 1.4 | (0.75–2.52) | | 0.31 | | 0.9 | (0.59–1.46) | | 0.76 | |
| No | 1.0 | . | | . | 1.0 | . | | . | | 1.0 | . | | . | |
| **Bilateral disease** |  |  | |  |  |  | |  | |  |  | |  | |
| Yes | 1.1 | (0.78–1.59) | | 0.56 | 2.0 | (1.19–3.53) | | **0.01** | | 1.8 | (1.17–2.77) | | **0.01** | |
| No | 1.0 | . | | . | 1.0 | . | | . | | 1.0 | . | | . | |
| **Resistance type** |  |  | |  |  |  | |  | |  |  | |  | |
| Primary | 1.0 | . | | . | 1.0 | . | | . | | 1.0 | . | | . | |
| Acquired | 1.5 | (0.98–2.29) | | **0.07** | 1.5 | (0.82–2.68) | | 0.19 | | 2.1 | (1.21–3.52) | | **0.01** | |
| **Initial resistance pattern** |  |  | |  |  |  | |  | |  |  | |  | |
| MDR/RR | 1.0 | . | | . | 1.0 | . | | . | | 1.0 | . | | . | |
| XDR | 0.8 | (0.09–6.55) | | 0.80 | 3.3 | (0.63–17.47) | | 0.16 | | 7.6 | (2.38–24.47) | | **<0.01** | |
| Initial scheme type |  |  | |  |  |  | |  | |  |  | |  | |
| Individualized | 1.3 | (0.77–2.22) | | 0.32 | 1.3 | (0.66–2.74) | | 0.42 | | 1.1 | (0.57–2.02) | | 0.82 | |
| Standardized | 1.0 | . | | . | 1.0 | . | | . | | 1.0 | . | | . | |
| **Fluoroquinolone treatment** |  |  | |  |  |  | |  | |  |  | |  | |
| Only used Lfx or Mfx | 1.0 | . | | . | 1.0 | . | | . | | 1.0 | . | | . | |
| Only used Ofx | 1.9 | (0.46–7.60) | | 0.38 | 2.8 | (0.56–14.28) | | 0.21 | | 0.9 | (0.10–7.35) | | 0.90 | |
| Used more than one fluorquinolone or did not use | 0.4 | (0.08–1.54) | | 0.17 | 1.6 | (0.54–4.84) | | 0.39 | | 1.5 | (0.59–3.80) | | 0.39 | |
| **Injectable drug treatment** |  |  | |  |  |  | |  | |  |  | |  | |
| Just used Am | 1.0 | . | | . | 1.0 | . | | . | | 1.0 | . | | . | |
| Only used S | 1.0 | (0.65–1.46) | | 0.88 | 0.9 | (0.51–1.69) | | 0.82 | | 0.8 | (0.54–1.33) | | 0.46 | |
| Only used Cm | 1.2 | (0.55–2.72) | | 0.61 | 1.5 | (0.51–4.32) | | 0.47 | | 0.6 | (0.20–1.85) | | 0.38 | |
| Used more than one injectable or did not use | 0.7 | (0.31–1.49) | | 0.33 | 2.4 | (1.07–5.20) | | **0.03** | | 1.0 | (0.48–2.18) | | 0.94 | |
| Block III |  |  | |  |  |  | |  | |  |  | |  | |
| **Resides in different municipality than treatment** |  |  | |  |  |  | |  | |  |  | |  | |
| Yes | 0.7 | (0.47–0.96) | | **0.03** | 1.3 | (0.79–2.05) | | 0.32 | | 0.8 | (0.55–1.22) | | 0.33 | |
| No | 1.0 | . | | . | 1.0 | . | | . | | 1.0 | . | | . | |
| **Reported unfavorable clinical evaluation** |  |  | |  |  |  | |  | |  |  | |  | |
| Yes | 3.1 | (2.07–4.73) | | **<0.01** | 15.8 | (9.19–27.08) | | **<0.01** | | 3.5 | (2.23–5.51) | | **<0.01** | |
| No | 1.0 | . | | . | 1.0 | . | | . | | 1.0 | . | | . | |
| **Changed regimen type** |  |  | |  |  |  | |  | |  |  | |  | |
| Yes | 0.5 | (0.32–0.94) | | **0.03** | 1.3 | (0.70–2.26) | | 0.45 | | 0.5 | (0.28–0.98) | | **0.04** | |
| No | 1.0 | . | | . | 1.0 | . | | . | | 1.0 | . | | . | |
| **Had adverse reaction** |  |  | |  |  |  | |  | |  |  | |  | |
| No adverse reaction records | 1.0 | . | | . | 1.0 | . | | . | | 1.0 | . | | . | |
| Only minor adverse reactions | 0.4 | (0.22–0.71) | | **<0.01** | 1.2 | (0.67–2.14) | | 0.54 | | 0.5 | (0.29–0.98) | | **0.04** | |
| At least one major adverse reaction | 0.5 | (0.21–1.06) | | **0.07** | 0.3 | (0.08–1.42) | | 0.14 | | 0.8 | (0.35–1.68) | | 0.52 | |
| Block IV |  |  | |  |  |  | |  | |  |  | |  | |
| **Previous TB events** |  |  | |  |  |  | |  | |  |  | |  | |
| Up to 3 events | 1.0 | . | | . | 1.0 | . | | . | | 1.0 | . | | . | |
| 4 events or more | 1.9 | (1.17–3.24) | | **0.01** | 0.9 | (0.38–2.21) | | 0.85 | | 1.9 | (1.06–3.36) | | **0.03** | |
| **Time between the first TB diagnosis and the onset of DR-TB treatment (years)** |  |  | |  |  |  | |  | |  |  | |  | |
| Up to 1 | 1.0 | . | | . | 1.0 | . | | . | | 1.0 | . | | . | |
| 1 to 3 | 1.0 | (0.68–1.57) | | 0.88 | 0.8 | (0.46–1.52) | | 0.56 | | 0.9 | (0.56–1.58) | | 0.83 | |
| More than 3 | 0.9 | (0.59–1.37) | | 0.63 | 0.8 | (0.47–1.49) | | 0.54 | | 1.5 | (0.93–2.27) | | 0.10 | |

OR: odds ratio

CI: confidence interval

*Significance level = 0.10

^1^ Brown/Black = combines black and brown

^2^ silicosis. neoplasms. transplant. user of TNF–alpha inhibitors and corticosteroids, seizure, viral hepatitis, renal failure/hemodialysis, and mental disorder

MDR: multidrug resistant

RR: resistant to rifampicin diagnosed by GeneXpert MTB/RIF® (RR)

XDR: extensively resistant

Lfx: levofloxacin

Mfx: moxifloxacin

Ofx: ofloxacino

Am: amikacin

S: streptomycin

Cm: capreomycin

TB: tuberculosis

Table S4: Table with goodness-of-fit test for a final multinomial logistic regression model result.

| Group | Prob | Obs_3 | Exp_3 | Obs_2 | Exp_2 | Obs_1 | Exp_1 | Obs_0 | Exp_0 | Total |
| --- | --- | --- | --- | --- | --- | --- | --- | --- | --- | --- |
| 1 | 0.1539 | 3 | 2.44 | 3 | 2.67 | 7 | 6.46 | 85 | 86.43 | 98 |
| 2 | 0.1914 | 6 | 4.87 | 4 | 3.27 | 8 | 8.81 | 80 | 81.06 | 98 |
| 3 | 0.2271 | 3 | 5.87 | 4 | 3.69 | 8 | 11.62 | 86 | 79.83 | 101 |
| 4 | 0.2685 | 7 | 7.85 | 2 | 3.51 | 14 | 12.43 | 72 | 71.21 | 95 |
| 5 | 0.3154 | 15 | 9.19 | 1 | 3.44 | 17 | 16.05 | 65 | 69.31 | 98 |
| 6 | 0.3672 | 8 | 12.94 | 2 | 3.45 | 25 | 16.98 | 63 | 64.64 | 98 |
| 7 | 0.4387 | 16 | 15.81 | 6 | 3.84 | 17 | 19.62 | 59 | 58.72 | 98 |
| 8 | 0.5416 | 17 | 16.53 | 11 | 8.47 | 20 | 23.52 | 50 | 49.47 | 98 |
| 9 | 0.6920 | 20 | 17.59 | 17 | 19.90 | 20 | 23.48 | 41 | 37.03 | 98 |
| 10 | 0.8932 | 25 | 26.91 | 26 | 23.76 | 27 | 24.02 | 20 | 23.31 | 98 |

| Number of observations | 980 |
| --- | --- |
| Number of outcome values | 4 |
| Base outcome value | 0 |
| Number of groups | 10 |
| Chi-squared statistic | 22.516 |
| Degrees of freedom | 24 |
| Prob > chi-squared | 0.549 |
